# Novel *ALG13* Variants and an Expanded Neurodevelopmental Spectrum: Genotype–Phenotype Correlations

**DOI:** 10.1155/humu/6800099

**Published:** 2026-07-26

**Authors:** Song Su, Wandong Hu, Ying Ren, Tong Zhang, Chunmei Yu, Hongwei Zhang

**Affiliations:** ^1^ Neurology Department, Children′s Hospital Affiliated to Shandong University, Jinan, Shandong, China; ^2^ Neurology Department, Jinan Children′s Hospital, Jinan, Shandong, China

**Keywords:** *ALG13* gene, congenital disorders of glycosylation, developmental and epileptic encephalopathy, genotype–phenotype, organoids

## Abstract

**Background:**

The *ALG13* gene is implicated in congenital disorders of glycosylation (CDG) and developmental and epileptic encephalopathy (DEE), yet genotype–phenotype correlations remain incompletely understood.

**Methods:**

Whole‐exome sequencing (WES) was performed in unrelated families, and we systematically reviewed existing patient data on *ALG13* variants and investigated the expression patterns of *ALG13* using organoid models.

**Results:**

This study reports five patients with ALG13 variants, including two novel variants. Four patients presented with epilepsy accompanied by neurodevelopmental impairment, including infantile epileptic spasms syndrome (IESS) and drug‐resistant epilepsy, whereas one patient showed developmental delay without seizures. Further analysis revealed that variants identified in patients with isolated developmental delay were primarily located outside the key glycosylation functional domain. Statistical analysis of patients with epileptic encephalopathy showed that infantile‐onset seizures were the predominant feature, with most cases being refractory to treatment. Additionally, in organoids, the highest *ALG13* expression was observed at 1 month in ectodermal‐derived neurons (EN). At 5 months, peak expression shifted to deep‐layer cortical neurons (EN‐CTX‐Deep).

**Conclusions:**

*ALG13* variants are associated with a broad phenotypic spectrum ranging from DEE to neurodevelopmental impairment without seizures. Our findings support a possible domain‐related genotype–phenotype association and provide additional developmental context for the role of *ALG13* in neurodevelopmental disorders. Further functional studies are required to clarify the pathogenic mechanisms of different *ALG13* variants.

## 1. Introduction

The *ALG13* gene plays an essential role in the biosynthesis of N‐linked glycans, which are crucial for the proper folding, trafficking, and function of glycoproteins. As part of the glycosylation pathway, *ALG13* is involved in adding a glycan moiety to nascent proteins, a process vital for their stability and functionality [1, 2]. *ALG13* encodes multiple protein isoforms. The long isoform (~1137 aa) contains an N‐terminal glycosyltransferase family 28 (GT28) domain, followed by predicted OTU‐like and Tudor‐like regions and an extended C‐terminal segment, which are thought to contribute to protein regulation and protein interactions. Importantly, biochemical studies indicate that the shorter ALG13 isoform which largely comprises the GT28 glycosyltransferase domain is sufficient to form a functional complex with ALG14. Functionally, ALG13 acts as the catalytic subunit of the UDP‐N‐acetylglucosamine transferase complex in the endoplasmic reticulum, working with ALG14 to catalyze an early step in the biosynthesis of dolichol‐linked oligosaccharides, a prerequisite for protein N‐glycosylation [3, 4]. Variants in *ALG13* have been associated with congenital disorders of glycosylation (CDG) and developmental and epileptic encephalopathy (DEE), which involve severe, often treatment‐resistant seizures and significant developmental regression, have also been recognized as a major clinical phenotype associated with *ALG13* variants [5, 6].

Despite the known connection between *ALG13* variants and neurological disorders, the precise molecular mechanisms linking these variants to clinical outcomes remain poorly understood [7]. While numerous studies have focused on the association between *ALG13* variants and epileptic encephalopathies, there is limited exploration of how variants in the *ALG13* gene may cause other neurodevelopmental phenotypes, such as isolated developmental delay. This creates a gap in the understanding of how specific variant sites or structural domains within *ALG13* contribute to different clinical presentations, especially given the essential role of glycosylation in neuronal development [8, 9]. We also explore the expression patterns of *ALG13* in brain organoids, focusing on how these patterns differ across developmental stages and in response to different variant sites.

Interestingly, clinical phenotyping has revealed that *ALG13* variants can lead to both epileptic encephalopathy and developmental delay but without the overt signs of significant glycosylation impairment that typically define CDG [5, 10]. While glycosylation markers in affected individuals may show subtle changes, the lack of striking glycosylation defects presents a puzzle. This suggests that the impact of *ALG13* variants on the nervous system may not always be directly linked to the extent of glycosylation disruption, but rather to the specific regions of the gene affected by variants and how these variants influence cellular processes in the brain [8, 9, 11–13]. Moreover, previous studies have primarily focused on variants associated with epileptic encephalopathy and glycosylation defects, with less attention given to those variants linked to isolated developmental delay [14]. In this study, we report five individuals carrying *ALG13* variants, including two novel missense variants, and systematically review previously reported cases to explore the clinical and genetic spectrum of *ALG13*‐related disorders.

## 2. Methods

### 2.1. Patients

Patients were recruited from a cohort of individuals with epilepsy and developmental delay at Children′s Hospital Affiliated to Shandong University. Inclusion criteria comprised infants and children diagnosed with epilepsy and/or developmental delay without acquired causes, such as structural brain lesions, metabolic disorders, tumors, trauma, infection, or stroke. Detailed clinical data were collected for each patient, including age at seizure onset, seizure types and frequency, family history, neurological examination findings, antiepileptic treatments, and prognosis. All patients underwent structural brain imaging using magnetic resonance imaging (MRI), which was independently reviewed by two experienced neuroradiologists, and reports were generated based on consensus. Long‐term (≥ 24 h) video electroencephalography (EEG) was performed using electrodes placed according to the international 10–20 reduced montage system. Standard procedures including eye‐opening/closing tests, hyperventilation, intermittent photic stimulation, and sleep recording were performed. EEG recordings were independently reviewed by at least two qualified electroencephalographers. Seizures and epilepsy syndromes were classified according to the International League Against Epilepsy (ILAE) guidelines (2022) [15–19].

All procedures were approved by the Ethics Committee of the participating hospitals, and written informed consent was obtained from all patients or their legal guardians.

### 2.2. Whole‐Exome Sequencing

Peripheral blood samples were collected from the proband and her parents into EDTA‐containing tubes for genomic DNA extraction using the QIAamp DNA Blood Midi Kit (Qiagen, Shanghai, China). DNA concentration and purity were assessed with a NanoDrop 2000 spectrophotometer (Thermo Fisher, United States). Clinical exome sequencing was performed on the NovaSeq 6000 platform (Illumina, United States) using the GenCap MedE006 capture kit (MyGenostics, Beijing, China). Paired‐end sequencing with 100 bp reads was conducted, achieving an average coverage depth of 100–150× and over 98% coverage of the target exotic regions. Sequencing reads were aligned to the human reference genome GRCh37 (hg19) using standard bioinformatic pipelines [20]. Single‐nucleotide variants (SNVs) and small insertion/deletion variants (indels) were called and annotated using the Genome Analysis Toolkit (GATK), following established protocols. Identified variants were further analyzed in the context of family segregation, population frequency databases, and in silico predictions to evaluate potential pathogenicity [21].

### 2.3. Genetic Analysis

Variants were annotated and functionally interpreted using ANNOVAR (http://annovar.openbioinformatics.org/en/latest/) and filtered against population databases including 1000 Genomes, ExAC, Genome Aggregation Database (gnomAD), ESP6500, and an in‐house database, excluding variants with suballelic frequency > 5*%*. Candidate variants were prioritized based on their relevance to the proband′s Human Phenotype Ontology (HPO) terms, a standardized nomenclature for systematically describing and classifying clinical phenotypes. The potential pathogenicity of novel variants was assessed using in silico prediction tools, including SIFT (v6.2.1, https://sift.bii.a-star.edu.sg/), PolyPhen‐2 (v2.2.2, http://genetics.bwh.harvard.edu/pph2/), MutationTaster (v2021, http://www.mutationtaster.org/), SpliceAI (v1.3.1, https://spliceailookup.broadinstitute.org/), and REVEL (v1.3, https://sites.google.com/site/revelgenomics/). Additionally, previously reported variants were evaluated through the Human Gene Mutation Database (HGMD) and ClinVar. All variants were classified according to the 2015 American College of Medical Genetics and Genomics and the Association for Molecular Pathology (ACMG/AMP) guidelines, and suspected pathogenic variants were further confirmed by Sanger sequencing in the proband and her parents for segregation analysis [22]. The functional impact of identified variants and their correlation with the patient′s clinical phenotype were evaluated using OMIM, dbSNP, Decipher, and relevant literature. Variant frequencies were compared with multiple control populations, including the East Asian and general populations in the gnomAD.

### 2.4. Damaging Effect of *ALG13* Variants

Structural modeling of both wild‐type and mutant proteins was carried out utilizing the SWISSMODEL online tool (http://www.swissmodel.expasy.org). The resulting protein structures were visualized and analyzed with PyMOL to examine spatial alterations, hydrogen bonding, and potential structural disruption induced by missense substitutions. Changes in protein stability resulting from single‐amino acid substitutions were further predicted using the I‐Mutant Suite, which calculates the free energy change (*Δ* *Δ* *G*, kcal/mol) [23]. Together, these analyses provided complementary insights into the potential pathogenicity of *ALG13* missense variants by integrating sequence conservation, predicted structural effects, and protein stability changes. The Varsite web server was used to analyze the changes in the hydrophobicity of each amino acid on the basis of the Fauch′ere and Pliska hydrophobicity scale [24]. The potential pathogenicity of each residue was further evaluated using the alpha missense score, which estimates the likelihood that a missense substitution at that position would be deleterious [25]. The p.K68E variant was introduced based on the WT model, and both WT and mutant structures were visualized in UCSF ChimeraX (v1.11).

### 2.5. Genotype–Phenotype Correlation Analysis

To investigate the relationship between genotype and phenotype, all pathogenic *ALG13* variants with definitive evidence were systematically reviewed and analyzed using data from the PubMed database and the professional edition of the HGMD up to December 2025. To further analyze the genotype–phenotype correlation, the information on variant types and the distributions of missense variants in *ALG13* were collected and analyzed.

### 2.6. *ALG13* Temporal Expression Profile Analysis

The general temporal expression profiles of *ALG13* at different developmental stages, including embryoid body, blastocyst, fetus, neonate, infant, juvenile, and adult stages, were retrieved from the UniGene database. Specifically, to analyze the temporal expression pattern in the brain, the *ALG13* expression in multiple brain areas at different developmental stages (from eight postconceptional weeks to 40 years) was obtained from the human RNA‐sequencing data in the BrainSpan database (http://www.brainspan.org/). For BrainSpan, RPKM‐normalized expression values across cortical regions were plotted against age, and expression trajectories were fitted using nonlinear regression (third‐order polynomial) in GraphPad Prism. Temporal expression patterns were compared with the observed seizure onset age in affected individuals.

### 2.7. Single‐Cell Expression Analysis of *ALG13* Genes

The cell‐type–specific expression of *ALG13* in the human brain was analyzed using publicly available single‐nucleus RNA sequencing (snRNA‐seq) data. Undirected cerebral organoid snRNA‐seq datasets were retrieved, and nuclei were classified into major neuronal and nonneuronal cell types according to canonical markers. *ALG13* expression levels across excitatory neurons, inhibitory neurons, and glial cell populations were subsequently assessed.

## 3. Results

### 3.1. Identification of *ALG13* Variants

**Figure 1:**
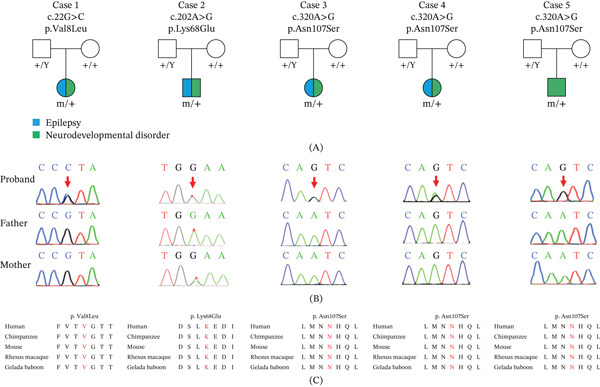
Genetic data of the cases with *ALG13* variants. (A) Pedigrees of the cases with *ALG13* variants and the corresponding phenotypes. (B) DNA sequencing chromatograms of the identified *ALG13* variants. Red arrows indicate the positions of the variants. (C) Amino acid sequence alignment showed that the identified missense variants are located in residues with highly on served across mammals.

In this study, five patients with *ALG13* variants were identified. Two patients carried previously unreported variants in *ALG13* (c.22G>C, p.Val8Leu and c.202A>G, p.Lys68Glu), and three patients harbored the recurrent hotspot variant (c.320A>G, p.Asn107Ser) (Figure 1A,B). Clinically, four patients were diagnosed with DEE, whereas one patient presented with isolated developmental delay. Notably, both patients with the two novel variants exhibited DEE, expanding the mutational spectrum associated with *ALG13*‐related DEE. The two novel variants were not recorded in the published literature or major clinical variant databases and were absent from population reference datasets (gnomAD v2.1.1, v3.1.2, and v4.1.0), with no hemizygous or homozygous carriers identified. Moreover, all affected residues were highly conserved across species (Figure 1C). Multiple in silico prediction tools (PolyPhen‐2, SIFT, MutationTaster, and CADD) supported a deleterious effect. Segregation analysis showed that four of the five patients carried de novo variants. Considering the de novo status together with rarity in population databases and concordant deleterious computational predictions, these variants were classified as likely pathogenic (LP) or pathogenic according to ACMG/AMP criteria. In contrast, Case 2 harbored a maternally inherited variant; although this variant was absent from gnomAD, mapped within a key functional domain, and was predicted to be damaging, it was classified as a variant of uncertain significance (VUS) (Table 1). Among the three patients carrying the p.Asn107Ser variant, two exhibited DEE, while one presented with isolated developmental delay, consistent with prior reports indicating phenotypic variability for this hotspot variant. No additional pathogenic variants were detected in other epilepsy‐associated genes in these patients, supporting ALG13 as the most plausible genetic contributor to the observed phenotypes.

**Table 1:** Genetic interpretation and predicted structural effects of *ALG13* variants identified in this cohort.

Case ID	Variant (NM_001099922.3)	Inheritance	gnomAD MAF	gnomAD EAS	DDG (kcal/mol)	Changes of hydrogen bonds	Changes of hydrophobicity	Alpha missense	Location	ACMG
1	c.22G>C	De novo	0	0	−1.72	Yes	No	0.946	GT28	LP (PS2 + PM2 + PP3)
	p.Val8Leu									
2	c.202A>G	Maternal	0	0	−0.43	No	No	0.073	GT28	VUS (PM2 + PP3)
	p.Lys68Glu									
3	c.320A>G	De novo	0	0	−0.51	No	No	0.622	GT28	P (PS2 + PS4 + PM2 + PM5 + PP3)
	p.Asn107Ser									
4	c.320A>G	De novo	0	0	−0.51	No	No	0.622	GT28	P (PS2 + PS4 + PM2 + PM5 + PP3)
	p.Asn107Ser									
5	c.320A>G	De novo	0	0	−0.51	No	No	0.622	GT28	P (PS2 + PS4 + PM2 + PM5 + PP3)
	p.Asn107Ser									

Abbreviations: ACMG/AMP, American College of Medical Genetics and Genomics/Association for Molecular Pathology; DDG or *Δ* *Δ* *G*, change in Gibbs free energy; EAS, East Asian; gnomAD, Genome Aggregation Database; GT28, glycosyltransferase 28 domain; LP, likely pathogenic; MAF, minor allele frequency; P, pathogenic; VUS, variant of uncertain significance.

### 3.2. Clinical Features of the Patients

The clinical characteristics of the five individuals carrying *ALG13* missense variants are summarized in Table 2. The cohort included three female patients and two male patients, with ages ranging from 3 years to 7 years and 9 months at the last follow‐up. Four patients presented with epilepsy, whereas one patient showed developmental delay without seizures. Seizure onset in most cases occurred in infancy, predominantly presenting as infantile epileptic spasms syndrome (IESS). All five patients had varying degrees of developmental delay or intellectual disability. Brain MRI was normal in all patients, and no major congenital malformations were observed. Liver function and coagulation profiles were within normal ranges in all cases.

**Table 2:** Clinical features of the cases with *ALG13* variants.

Case ID	Variant (NM_001099922.3)	Inheritance	Sex	Age	Seizure onset	Seizure type	EEG	MRI	ASMs	Seizure‐free	Liver function/coagulation	Developmental delay	Epilepsy syndrome
1	c.22G>C	De novo	F	3 years	15 days	ES	Hypsarrhythmia	Normal	ACTH	No	Normal	Severe DD, ID	West
	p.Val8Leu								TPM				
									KD				
2	c.202A>G	Maternal	M	7 years 9 months	7 years 3 months	CPS	Right frontopolar/frontal and midline (Fz) spike sharp waves; 2–3 Hz spike and slow sharp‐and‐slow wave complexes	Normal	OXC	No	Normal	Severe DD, ID	DEE
	p.Lys68Glu												
3	c.320A>G	De novo	F	6 months 13 days	2 months	ES	Hypsarrhythmia	Normal	ACTH	No	Normal	Severe DD, ID	West
	p.Asn107Ser								TPM				
									VPA				
4	c.320A>G	De novo	F	5 years 6 months	6 months	ES	6m: hypsarrhythmia; current: generalized sharp waves, spike‐and‐wave complexes, and spike‐and‐wave discharges	Normal	VPA	No	Normal	Severe DD, ID	West→LGS
	p.Asn107Ser					TS			TPM				
						AAS			PER				
5	c.320A>G	De novo	M	6 years	—	—	—	Normal	—	—	Normal	Mild DD, ID	—
	p.Asn107Ser												

Abbreviations: AAS, atypical absence seizure; ACTH, adrenocorticotropic hormone; ASMs, antiseizure medications; CPS, complex partial seizure; DD, developmental delay; DEE, developmental and epileptic encephalopathy; EEG, electroencephalography; ES, epileptic spasms; F, female; ID, intellectual disability; KD, ketogenic diet; LGS, Lennox–Gastaut syndrome; M, male; MRI, magnetic resonance imaging; OXC, oxcarbazepine; PER, perampanel; TPM, topiramate; TS, tonic seizure; VPA, valproic acid; West, West syndrome.

**Figure 2:**
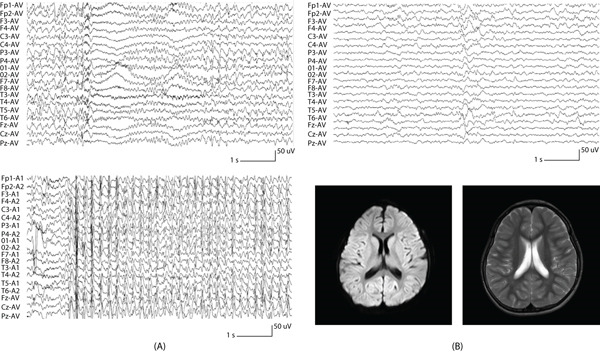
(A) Representative ictal EEG recordings. Case 1, epileptic spasms with generalized epileptiform activity. Case 2, focal seizure with focal epileptiform discharges. Case 4, atypical absence seizure with generalized spike‐and‐wave/slow spike‐and‐wave activity. (B) No structural abnormalities were observed on cranial MRI in the patient cohort.

Case 1 was a 3‐year‐old girl. She developed epileptic spasms at 15 days of age and was diagnosed with West syndrome. EEG showed hypsarrhythmia (Figure 2A), whereas brain MRI was normal. She was treated with ACTH, topiramate, and ketogenic diet; however, seizures remained uncontrolled. She showed severe developmental delay and intellectual disability. Case 2 developed seizures at 7 years and 3 months of age and presented with focal seizures. EEG showed right frontopolar/frontal and midline epileptiform discharges (Figure 2A). Brain MRI was normal. He was treated with oxcarbazepine, but seizures were not fully controlled. He had severe developmental delay and intellectual disability. Case 3 developed epileptic spasms at 2 months of age and was diagnosed with West syndrome. EEG revealed hypsarrhythmia. She received ACTH, topiramate, and valproate, but seizures remained refractory. Case 4 developed epileptic spasms at 6 months of age and subsequently developed multiple seizure types, including tonic seizures and atypical absence seizures. Her epilepsy syndrome evolved from West syndrome to Lennox–Gastaut syndrome (LGS). EEG initially showed hypsarrhythmia at 6 months and later demonstrated generalized sharp waves, spike‐and‐wave complexes, and spike‐and‐wave discharges (Figure 2A). She received valproate, topiramate, and perampanel. Case 5, unlike the other patients, did not develop seizures during follow‐up. He presented with mild developmental delay and intellectual disability. Brain MRI was unremarkable in all five patients, with no structural abnormalities detected (Figure 2B). Regarding treatment, all four patients with epilepsy developed drug‐resistant epilepsy, requiring multiple adjustments of antiseizure medications. All five patients displayed varying degrees of developmental delay, involving language, motor, and cognitive domains. Routine biochemical and ancillary investigations were within the normal range.

### 3.3. Genotype–Phenotype Analysis of *ALG13* Variants

**Figure 3:**
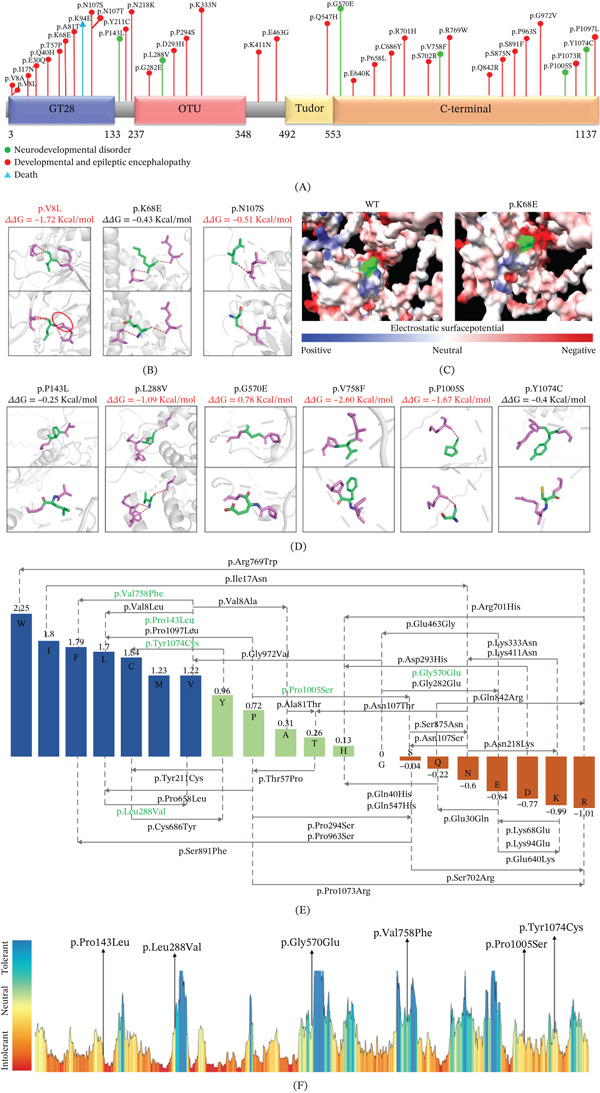
Molecular locations, damaging effect, and genotype–phenotype correlation of *ALG13* variants. (A) Schematic representation of the *ALG13* protein with annotated domains and the positions of missense variants identified in this study and those reported previously. Variant labels are color‐coded by clinical phenotype. (B) Representative structural modeling of variants from the current cohort (p.Val8Leu, p.Lys68Glu, and p.Asn107Ser), showing local interaction changes and red dashed lines indicate hydrogen bonds. Predicted *Δ* *Δ* *G* values are shown. (C) Electrostatic potential mapped onto the molecular surface for WT (K68) and p.K68E around Residue 68. (D) Structural modeling of six missense variants reported in individuals with isolated developmental delay (p.Pro143Leu, p.Leu288Val, p.Gly570Glu, p.Val758Phe, p.Pro1005Ser, and p.Tyr1074Cys), with predicted *Δ* *Δ* *G* values and hydrogen‐bond assessment. (E) Substituted residues mapped onto the Fauchère–Pliska hydrophobicity scale to illustrate physicochemical changes. (F) Regional residue‐level tolerance/intolerance landscape across *ALG13* with the positions of the six isolated developmental delay–associated variants. GT28, glycosyltransferase family 28 domain; OTU, ovarian tumor domain; Tudor, Tudor domain.

The N‐terminal GT28/glycosyltransferase‐related domain of *ALG13* was annotated according to the NCBI Conserved Domain Database, which defines the Glycosyltransferase_GTB_type region at amino acids 3–133 of *ALG13*. We compared the clinical features of our five patients with *ALG13* variants with previously published *ALG13*‐related cases and mapped all reported variants onto the protein domain architecture (Figure 3A) [5, 10, 14, 26–50]. Two novel missense variants identified in this study (c.22G>C, p.Val8Leu and c.202A>G, p.Lys68Glu) are located within the glycosylation‐critical functional region, and the associated phenotypes were concordant with the severe neurological manifestations typically reported for variants affecting this region, namely, DEE. Case 1, carrying p.Val8Leu, presented with DEE and showed marked predicted functional impact, including a pronounced reduction in protein stability, altered hydrogen‐bonding patterns, and an increased AlphaMissense score, consistent with a deleterious effect (Table S1). Case 2, carrying p.Lys68Glu, had a later seizure onset in the school‐age period with refractory focal epilepsy accompanied by severe developmental delay. Although structural metrics (protein stability and hydrogen bonding) were not substantially altered (Figure 3B), the variant′s localization within the glycosylation‐critical region supports a pathogenic contribution to a severe neurodevelopmental phenotype. Importantly, this variant also leads to an electrostatic charge reversal at Position 68, where the positively charged lysine is replaced by the negatively charged glutamic acid (Figure 3C and Figure S1). In contrast, our literature review identified six individuals reported with isolated developmental delay as the predominant phenotype; notably, the corresponding variants were all located outside the glycosylation‐critical region. Conversely, a previously reported patient with CDG including a fatal outcome harbored a variant within the glycosylation‐critical region. Collectively, these observations support a domain‐dependent genotype–phenotype relationship in *ALG13* which variants within the glycosylation‐critical region tend to be associated with more severe outcomes, whereas isolated developmental delay can occur and appears to cluster outside this key functional region.

### 3.4. Damage Prediction and Structural Analysis of *ALG13* Variants

Structural and in silico analyses of published *ALG13* variants associated with an isolated developmental delay phenotype identified six missense changes including c.428C>T (p.Pro143Leu), c.862C>G (p.Leu288Val), c.1709G>A (p.Gly570Glu), c.2272G>T (p.Val758Phe), c.3013C>T (p.Pro1005Ser), and c.3221A>G (p.Tyr1074Cys). Notably, all six variants were located outside the glycosylation‐critical functional domain (Figure 3A). Structural modeling did not predict alterations in hydrogen‐bonding networks for any of these substitutions (Figure 3D). Hydrophobicity profiling further indicated no appreciable hydrophobicity changes for p.Leu288Val, p.Gly570Glu, and p.Val758Phe. For the remaining three variants, polarity changes were predicted but appeared modest (Figure 3E). Overall, tolerance‐based in silico predictions suggested that all six substitutions were tolerated (Figure 3F), and AlphaMissense classified five variants (p.Pro143Leu, p.Gly570Glu, p.Val758Phe, p.Pro1005Ser, and p.Tyr1074Cys) as likely benign, whereas p.Leu288Val yielded an uncertain prediction (Table S1). Collectively, these findings suggest that variants associated with isolated developmental delay tend to cluster in nonglycosylation regions and are predicted to cause at most subtle functional perturbations.

### 3.5. Spatiotemporal Expression of *ALG13*

**Figure 4:**
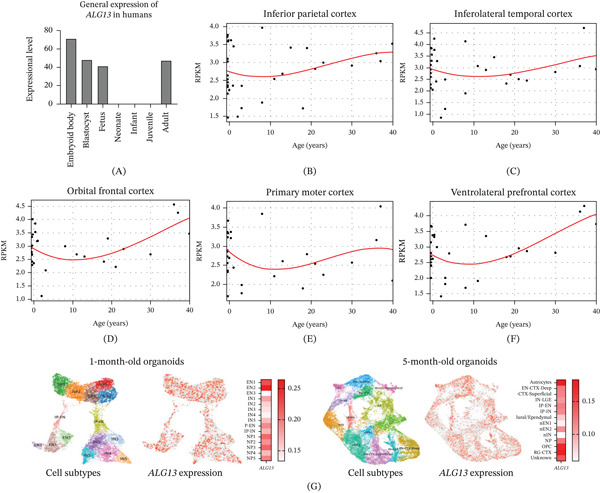
The temporal expression profile of *ALG13* in humans. (A) Temporal expression pattern of *ALG13* in humans retrieved from the UniGene database. (B–F) Temporal expression patterns of *ALG13* in different cortices of the human brain retrieved from the BrainSpan database. RPKM, reads per kilobase per million mapped read. (G) The single‐cell expression data of *ALG13* was retrieved from 1‐month and 5‐month undirected cerebral organoids.

To explore the relationship between *ALG13* gene expression and the observed clinical phenotypes, we analyzed its spatiotemporal expression using publicly available transcriptomic data. *ALG13* exhibited broad expression across multiple brain regions, particularly within the cortex and subcortex. Developmentally, *ALG13* expression was relatively high during fetal development, with notable peaks during the neonatal and infant stages, corresponding to critical periods of brain development (Figure 4A). This early high expression pattern aligns with the early onset of epileptic encephalopathy, as seen in patients with infantile‐onset seizures. Following birth, however, expression in the brain gradually decreased. Interestingly, expression of *ALG13* in brain regions increased again during later developmental stages, particularly in adult brain regions like the inferior parietal cortex, orbital frontal cortex, and ventrolateral prefrontal cortex (Figure 4B–F). This resurgence of expression in later stages corresponds with the clinical trend of drug‐resistant epilepsy observed in many patients, which may be related to the functional maturation of the brain areas involved in seizure control and motor function.

### 3.6. Expression Dynamics in Brain Organoids

At 1 month of age, *ALG13* expression was primarily observed in ectodermal‐derived neurons (EN) and inhibitory progenitor cells (IP‐IN), which are crucial for early neuronal development and cortical organization. This high expression in early developmental stages likely contributes to the early onset of epileptic encephalopathy and neurodevelopmental impairments. By 5 months of age, *ALG13* expression shifted toward deep‐layer cortical neurons (EN‐CTX‐Deep) and superficial cortical neurons (CTX‐Superficial), which play key roles in cognitive function, motor control, and sensory processing (Figure 4G). This increased expression in later developmental stages aligns with the observed delayed cognitive and motor functions in patients, particularly those with drug‐resistant epilepsy. These findings suggest that *ALG13* may significantly influence both early and late neurodevelopmental processes, contributing to the severity of clinical phenotypes observed in *ALG13*‐related disorders.

## 4. Discussion

The results of this study provide valuable insights into the clinical and molecular features of *ALG13* variants and their role in neurodevelopmental disorders. By identifying two novel missense variants in *ALG13*, we expanded the variant spectrum of this gene and further explored genotype–phenotype associations in *ALG13*‐related epileptic encephalopathy and developmental delay. Our findings suggest that *ALG13* variants are associated with a broad phenotypic spectrum, ranging from severe DEE to milder neurodevelopmental impairment without seizures. This differential clinical presentation may be influenced by the specific variant site within *ALG13* and the potential functional consequences of the variant on protein stability, local molecular interactions, and neuronal development.

The recurrent p.Asn107Ser variant remains the best established *ALG13* variant associated with DEE, particularly IESS. In our cohort, two patients carrying p.Asn107Ser presented with West syndrome, and one subsequently evolved into Lennox–Gastaut syndrome, consistent with the severe epileptic phenotype previously associated with this recurrent variant. In addition, we identified two novel missense variants, c.22G>C (p.Val8Leu) and c.202A>G (p.Lys68Glu), both located within the annotated N‐terminal GT28/glycosyltransferase‐related functional region. Case 1 carrying p.Val8Leu presented with early‐onset DEE, and in silico/structural analyses suggested a potential functional impact. Case 2 carrying p.Lys68Glu had school‐age onset drug‐resistant focal epilepsy accompanied by marked developmental delay, indicating that variants affecting this key functional region may contribute to severe neurological impairment even when seizure onset is later. In contrast, our literature synthesis suggests that variants reported in individuals with isolated developmental delay tend to be located outside the glycosyltransferase‐related functional region and may be associated with comparatively mild molecular perturbations, supporting a domain‐influenced genotype–phenotype relationship in *ALG13*‐related disorders.

Structural modeling and in silico analyses suggested heterogeneous molecular consequences among the *ALG13* variants identified in our cohort. For p.Val8Leu, multiple computational lines of evidence supported a substantial functional impact, including reduced predicted protein stability, altered local interaction networks, and an elevated AlphaMissense score, consistent with a deleterious effect and concordant with the severe early‐onset DEE phenotype. The p.Lys68Glu variant was absent from population databases, located within a functionally important region, and introduced a charge reversal with a localized electrostatic change; however, structural modeling did not show marked destabilization or major hydrogen‐bond rearrangement. Moreover, the variant was maternally inherited and classified as a VUS. Although maternally inherited ALG13 variants may be associated with mild or atypical phenotypes, potentially related to variable expressivity, incomplete penetrance, skewed X‐chromosome inactivation, or modifying factors, the limited lack of functional validation indicates that p.Lys68Glu should be considered a candidate variant rather than definitive evidence of pathogenicity.

ALG13 is involved in N‐linked glycosylation through formation of the ALG13–ALG14 UDP‐GlcNAc transferase complex. Previous functional studies have reported abnormalities in glycosylation‐related readouts associated with ALG13 variants, including UDP‐GlcNAc transferase activity, cellular N‐glycan profiles, and glycoprotein‐related markers [10, 26]. Previous studies of ALG13‐CDG and ALG13‐related developmental disorders have mainly emphasized variants located within the GT28/glycosyltransferase‐related domain. In contrast, some variants associated with neurodevelopmental phenotypes in the present study and in the literature‐based analysis were located outside this canonical functional region. This observation suggests that the phenotypic impact of ALG13 may not be restricted to direct disruption of the catalytic core. Instead, variants outside the annotated GT28 domain may contribute to disease through alternative mechanisms, such as altered protein stability, impaired ALG13–ALG14 complex formation, disturbed subcellular localization, or effects on protein–protein interactions [3].

The analysis of spatiotemporal expression of *ALG13* in brain tissue revealed that the gene is widely expressed across various brain regions, with distinct peaks during critical periods of neurodevelopment, including fetal and neonatal stages, as well as during school‐age development. These findings are consistent with the age of seizure onset observed in patients, who presented with early‐onset epileptic encephalopathy. The timing of *ALG13* expression in specific brain regions likely plays a significant role in the observed seizure onset and developmental delay [7, 11]. Specifically, the higher expression of *ALG13* during fetal and early postnatal stages suggests its potential involvement in early brain development, while later peaks in expression align with the onset of generalized seizures in the affected individuals. These results suggest that the temporal dynamics of *ALG13* expression may influence both the timing of clinical symptom onset and the severity of the associated phenotypes, particularly in neurodevelopmental disorders.

Single‐cell RNA sequencing of human brain organoids showed that these temporal and cell‐type–specific patterns suggest that *ALG13* may be particularly relevant during early neuronal differentiation and later cortical circuit maturation. While transcriptomic enrichment alone cannot directly explain seizure types or developmental outcomes, the observed expression windows overlap with periods of heightened neurodevelopmental vulnerability and may help contextualize why *ALG13*‐related phenotypes range from infantile‐onset DEE to isolated developmental delay.

Our findings support the inclusion of ALG13 in the genetic evaluation of neurodevelopmental disorders, particularly in patients with infantile spasms, drug‐resistant DEE, and no definite epileptogenic lesion on MRI. In addition, the identification of a patient with isolated developmental delay without seizures highlights that *ALG13* should also be considered in nonepileptic neurodevelopmental presentations and that longitudinal surveillance for potential later‐onset seizures may be appropriate. Moreover, variant interpretation may benefit from integrating domain location, in silico evidence, and developmental expression context rather than relying on a single predictor or assuming a fixed phenotype. This study has several limitations, including the small number of newly identified patients, the retrospective and heterogeneous nature of the literature‐based analysis, incomplete glycosylation testing, and the lack of direct functional validation. In addition, interpretation of the maternally inherited p.Lys68Glu variant was limited by unavailable maternal clinical information.

In conclusion, this study expands the mutational spectrum of *ALG13* and provides important insights into the genotype–phenotype correlations associated with epileptic encephalopathy and isolated developmental delay. Our findings suggest that *ALG13* variants outside the glycosylation domain lead to milder, epilepsy‐only phenotypes, while variants within the critical glycosylation domain cause more severe multisystem involvement. The temporal and cell‐type–specific expression patterns of *ALG13* likely contribute to the observed clinical variability, emphasizing the need for personalized management strategies based on the variant site and age of onset.

## Author Contributions

S.S. contributed to the study conception and design. Y.R. and T.Z. collected the clinical data. S.S. and W.H. performed the bioinformatics prediction. The first draft of the manuscript was written by S.S. Manuscript editing and manuscript review were carried out by H.Z. and C.Y. S.S. and W.H. are first authors.

## Funding

This study was supported by the Third Batch of National Demonstration Projects for Public Hospital Reform and High‐Quality Development.

## Disclosure

All authors contributed to the article and approved the submitted version. We confirm that we have read the journal′s position on issues involved in ethical publication and affirm that this report is consistent with those guidelines.

## Ethics Statement

This study involves human participants and was approved by an Ethics Committee(s) or Institutional Board(s). This work has been approved by the Ethics Committee of Children′s Hospital affiliated to Shandong University. All the procedures performed in the study were according to the Declaration of Helsinki.

## Conflicts of Interest

The authors declare no conflicts of interest.

## Supporting Information

Additional supporting information can be found online in the Supporting Information section.

## Supporting information


**Supporting Information 1** 

**Supporting Information 2** 

## Data Availability

The data that support the findings of this study are available from the corresponding authors upon reasonable request.
