# Supplementary material for: Novel ALG13 Variants and an Expanded Neurodevelopmental Spectrum: Genotype–Phenotype Correlations
Source: Hum Mutat. 2026 Jul 26;2026:6800099. doi: 10.1155/humu/6800099 (PMC13402893; doi:10.1155/humu/6800099)
Supplement: Supplementary file 1 — Supporting Information 1 [file HUMU-2026-6800099-s002.docx]

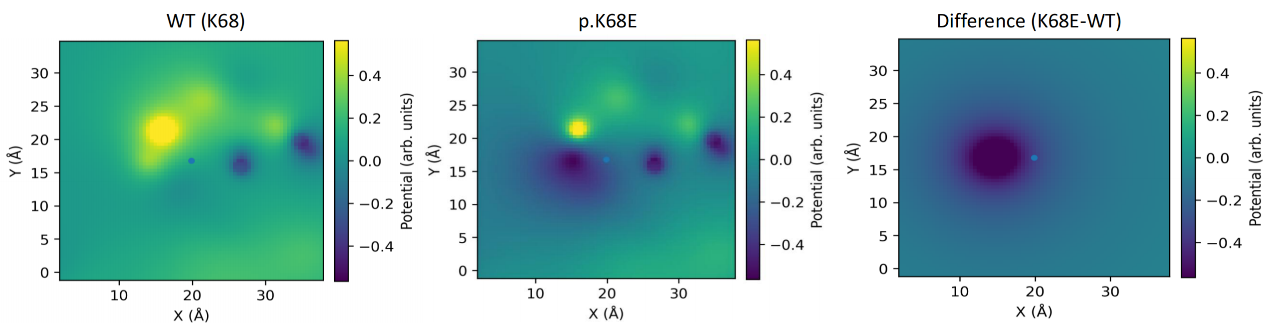


**Supplementary figure 1**: Electrostatic potential slice near residue 68 for WT (K68), p.K68E, and the difference map (K68E−WT).
