# Supplementary material for: Novel ALG13 Variants and an Expanded Neurodevelopmental Spectrum: Genotype–Phenotype Correlations
Source: Hum Mutat. 2026 Jul 26;2026:6800099. doi: 10.1155/humu/6800099 (PMC13402893; doi:10.1155/humu/6800099)
Supplement: Supplementary file 2 — Supporting Information 2 [file HUMU-2026-6800099-s001.docx]

| **ID** | **Variant** | **gnomAD MAF** | **gnomAD EAS** | **DDG (Kcal/mo)** | **Changes of**  **hydrogen bonds** | **Changes of**  **Hydrophobicity** | **Alphamissense** | **Mutation tolerance** | **Location** |
| --- | --- | --- | --- | --- | --- | --- | --- | --- | --- |
| 1 | c.23T>C  p.Val8Ala | 0 | 0 | -3.25 | Yes | Yes | 0.758 | 0.72 | GT28 |
| 2 | c.22G>C  p.Val8Leu | 0 | 0 | -1.72 | Yes | No | 0.946 | 0.72 | GT28 |
| 3 | c.50T>A  p.Ile17Asn | 0 | 0 | -4.31 | No | Yes | 0.969 | 0.62 | GT28 |
| 4 | c.88G>C  p.Glu30Gln | 0 | 0 | -0.54 | Yes | No | 0.067 | 0.46 | GT28 |
| 5 | c.120A>C  p.Gln40His | 0 | 0 | -0.99 | Yes | Yes | 0.983 | 0.54 | GT28 |
| 6 | c.169A>C  p.Thr57Pro | 0 | 0 | -1.36 | Yes | No | 0.353 | 0.5 | GT28 |
| 7 | c.202A>G  p.Lys68Glu | 0 | 0 | -0.43 | No | No | 0.073 | 0.77 | GT28 |
| 8 | c.241G>A  p.Ala81Thr | 0 | 0 | -0.84 | Yes | No | 0.921 | 0.31 | GT28 |
| 9 | c.280A>G  p.Lys94Glu | 0 | 0 | -1.27 | Yes | No | 0.901 | 0.39 | GT28 |
| 10 | c.320A>G  p.Asn107Ser | 0 | 0 | -0.51 | No | No | 0.622 | 0.5 | GT28 |
| 11 | c.320A>C  p.Asn107Thr | 0 | 0 | -0.33 | Yes | Yes | 0.818 | 0.5 | GT28 |
| 12 | c.428C>T  p.Pro143Leu | 0 | 0 | -0.25 | No | Yes | 0.083 | 0.28 | GT28-OUT  linker |
| 13 | c.632A>G  p.Tyr211Cys | 0 | 0 | 0.28 | No | Yes | 0.08 | 0.9 | GT28-OUT  linker |
| 14 | c.654T>G  p.Asn218Lys | 0 | 0 | 0.7 | Yes | No | 0.277 | 0.48 | GT28-OUT  linker |
| 15 | c.845G>A  p.Gly282Glu | 0.00001146 | 0 | 0.43 | Yes | No | 0.882 | 0.64 | OTU |
| 16 | c.862C>G  p.Leu288Val | 0.00001147 | 0 | -1.09 | No | No | 0.421 | 0.85 | OTU |
| 17 | c.877G>C  p.Asp293His | 0 | 0 | -0.18 | Yes | Yes | 0.68 | 0.63 | OTU |
| 18 | c.880C>T  p.Pro294Ser | 0.00006349 | 0 | -0.84 | No | Yes | 0.814 | 0.7 | OTU |
| 19 | c.999G>C  p.Lys333Asn | 0 | 0 | -0.54 | No | No | 0.881 | 0.23 | OTU |
| 20 | c.1233G>C  p.Lys411Asn | 0 | 0 | -0.54 | No | No | 0.248 | 0.56 | OUT- Tudor  linker |
| 21 | c.1388A>G  p.Glu463Gly | 0.00002318 | 0 | -1.16 | Yes | Yes | 0.232 | 0.11 | OUT- Tudor  linker |
| 22 | c.1641A>T  p.Gln547His | 0 | 0 | -0.86 | No | Yes | 0.259 | 0.96 | Tudor |
| 23 | c.1709G>A  p.Gly570Glu | 0 | 0 | 0.78 | No | Yes | 0.11 | 0.95 | C-terminal |
| 24 | c.1918G>A  p.Glu640Lys | 0 | 0 | 0.91 | No | No | 0.144 | 0.77 | C-terminal |
| 25 | c.1973C>T  p.Pro658Leu | 0.000009986 | 0 | 0.59 | No | Yes | 0.127 | 0.61 | C-terminal |
| 26 | c.2057G>A  p.Cys686Tyr | 0.000005678 | 0 | -2.32 | No | Yes | 0.122 | 0.38 | C-terminal |
| 27 | c.2102G>A  p.Arg701His | 0.000005957 | 0 | -1.68 | No | Yes | 0.102 | 0.87 | C-terminal |
| 28 | c.2106T>G  p.Ser702Arg | 0 | 0 | -0.86 | No | No | 0.836 | 0.93 | C-terminal |
| 29 | c.2272G>T  p.Val758Phe | 0.000005591 | 0 | -2.60 | No | No | 0.143 | 2.16 | C-terminal |
| 30 | c.2305C>T  p.Arg769Trp | 0.00002238 | 0 | -0.48 | No | Yes | 0.105 | 1.39 | C-terminal |
| 31 | c.2525A>G  p.Gln842Arg | 0 | 0 | -0.26 | No | No | 0.105 | 0.79 | C-terminal |
| 32 | c.2624G>A  p.Ser875Asn | 0.000005609 | 0 | 1.27 | No | No | 0.097 | 0.74 | C-terminal |
| 33 | c.2672C>T  p.Ser891Phe | 0.005472 | 0 | -0.45 | No | Yes | 0.172 | 0.83 | C-terminal |
| 34 | c.2887C>T  p.Pro963Ser | 0 | 0 | -1.43 | No | Yes | 0.14 | 0.47 | C-terminal |
| 35 | c.2915G>T  p.Gly972Val | 0 | 0 | -1.4 | No | Yes | 0.954 | 0.37 | C-terminal |
| 36 | c.3013C>T  p.Pro1005Ser | 0 | 0 | -1.67 | No | Yes | 0.15 | 0.42 | C-terminal |
| 37 | c.3218C>G  p.Pro1073Arg | 0.000005676 | 0 | -2.18 | No | Yes | 0.122 | 0.64 | C-terminal |
| 38 | c.3221A>G  p.Tyr1074Cys | 0.0006346 | 0 | -0.4 | No | Yes | 0.062 | 0.61 | C-terminal |
| 39 | c.3290C>T  p.Pro1097Leu | 0.000009984 | 0 | -1.38 | No | Yes | 0.083 | 0.77 | C-terminal |

Supplement table 1. Genetic characteristics, damaging effect of the *ALG13* variants.
